# Endocrine and molecular factors of increased female reproductive performance in the Dummerstorf high-fertility mouse line FL1

**DOI:** 10.1530/JME-22-0012

**Published:** 2022-04-06

**Authors:** Carolin Lisa Michaela Ludwig, Simon Bohleber, Alexander Rebl, Eva Katrin Wirth, Marzia Tindara Venuto, Martina Langhammer, Ulrich Schweizer, Joachim M Weitzel, Marten Michaelis

**Affiliations:** 1 Institute of Reproductive Biology, Research Institute for Farm Animal Biology (FBN), Dummerstorf, Germany; 2 Institut für Biochemie und Molekularbiologie (IBMB), Rheinische Friedrich-Wilhelms-Universität Bonn, Bonn, Germany; 3 Institute of Genome Biology, Fish Genetics Unit, Research Institute for Farm Animal Biology (FBN), Dummerstorf, Germany; 4 Department of Endocrinology and Metabolism, Charité – Universitätsmedizin Berlin, corporate member of Freie Universität Berlin, Humboldt-Universität zu Berlin, Berlin, Germany; 5 DZHK (German Centre for Cardiovascular Research), partner site Berlin, Berlin, Germany; 6 Institute of Genetics and Biometry, Service Group Model Laboratory Animals, Research Institute for Farm Animal Biology (FBN), Dummerstorf, Germany

**Keywords:** reproductive fitness, female high-fertility, folliculogenesis, ovulation rate, estrous cycle, HPG axis

## Abstract

The Dummerstorf high-fertility mouse line FL1 is a worldwide unique selection experiment for increased female reproductive performance. After more than 190 generations of selection, these mice doubled the amount of offspring per litter compared to the unselected control line. FL1 females have a superior lifetime fecundity and the highest Silver fecundity index that has been described in mice, while their offspring show no signs of growth retardation. The reasons for the increased reproductive performance remained unclear. Thus, this study aims to characterize the Dummerstorf high-fertility mouse line FL1 on endocrine and molecular levels on the female side. We analyzed parameters of the hypothalamic pituitary gonadal axis on both hormonal and transcriptional levels. Gonadotropin-releasing hormone and follicle-stimulating hormone (FSH) concentrations were decreased in FL1 throughout the whole estrous cycle. Luteinizing hormone (LH) was increased in FL1 mice in estrus. Progesterone concentrations were decreased in estrus in FL1 mice and not affected in diestrus. We used a holistic gene expression approach in the ovary to obtain a global picture of how the high-fertility phenotype is achieved. We found several differentially expressed genes in the ovaries of FL1 mice that are associated with different female fertility traits. Our results indicate that ovulation rates in mice can be increased despite decreased FSH levels. Cycle-related alterations of progesterone and LH levels have the potential to improve follicular maturation, and interactions of endocrine and molecular factors lead to enhanced follicular survival, more successful folliculogenesis and therefore higher ovulation rates in female FL1 mice.

## Introduction

Exploratory mouse models are used to gain insights into the biology of reproduction. In most of these models, genes are overexpressed or deleted to conclude their function in reproductive performance. In the database Mouse Genome Informatics, more than 1700 genotypes are annotated with a female fertility phenotype. The main proportions of these annotations refer to infertility (41%), subfertility (26%), and decreased litter size (31%). Only a tiny minority of genotypes are linked with enhanced fertility (0.3%) or increased litter size (1.8%) (http://www.informatics.jax.org/). Thus, most knowledge on reproduction is based on the adverse phenotype, and a lack of high-fertility models is striking. For this study, we completely changed the perspective using a worldwide unique outbred mouse model. The Dummerstorf high-fertility mouse line FL1 was selected for increased litter size and the total birth weight of the litters for more than 190 generations. This mouse line is neither a transgenic nor a knockout model. Today, after more than 45 years of selection, FL1 almost doubled the number of pubs per litter, along with the total birth weight of the entire litters compared to the unselected control line (ctrl) in the first parturition, with no signs of growth retardation in the offspring.

Previous data of a two-factorial crossbreeding experiment from our laboratory indicate that the improved fertility phenotype is mostly warranted on the female side (Langhammer *et al.* 2017). However, altered physiological parameters including testicular gene expression are also evident in FL1 vs ctrl males (Michaelis *et al.* 2013, 2018). Females of FL1 ovulate approximately twice as many oocytes compared to ctrl females (Spitschak *et al.* 2007). Remarkably, the FL1 females do not only deliver a high number of pubs in the first pregnancy, but they are also able to deliver large litter sizes with a high reproductive mating rate over a long-time period without health issues (Langhammer *et al.* 2021). Integrating these three parameters, FL1 females have by far the highest Silver fecundity index (the most reliable index for evaluating the reproductive performance of mouse strains) that has ever been described in literature (Silver 1995, Langhammer *et al.* 2021).

Histologic analysis reveals that the occurrence of polyovular follicles (POFs) is characteristic for FL1 females. Although POFs were observed in both FL1 and ctrl mice, they were much more frequent in FL1. Differences were also shown in the number of oocytes inside the POFs. In FL1 mice, the POFs contained up to seven oocytes per follicle, while POFs of ctrl mice contained a maximum of two oocytes per follicle (Alm *et al.* 2010).

Based on the results of previous studies, alterations in follicular development and/or an altered hypothalamic pituitary gonadal (HPG) axis activity are prime candidate pathways responsible for the increased ovulation phenotype.

The foundation for subsequent follicular development is the pool of non-growing primordial follicles, which are already developed around postnatal day 4 in mice (Pangas & Rajkovic 2015). During follicular development, transition from the primordial follicle to the growing preantral follicle occurs. The growing follicles achieve antral and finally preovulatory stage. Due to follicular atresia, only less than 1% of the initially formed primordial follicles succeed in entering maturation and ovulation (Bjersing 1982). Antral and preovulatory follicles are regulated by hormones of the HPG axis. Gonadotropin releasing hormone (GnRH) from the hypothalamus leads to pulsatile secretion of follicle-stimulating hormone (FSH) and luteinizing hormone (LH) in the pituitary, which both in turn modulate the secretion of ovarian steroid hormones. FSH is not essential for early follicular development, but it is required for antral follicle formation, ovulation, and the release of developmentally competent oocytes (Demeestere *et al.* 2012). LH is also needed for the formation of antral follicles and the induction of ovulation (Ma *et al.* 2004). Furthermore, a role of LH in preantral follicle development has been discussed in literature (Wu *et al.* 2000). Hence, alterations in the parameters of the HPG axis influence folliculogenesis and the number of ovulated oocytes.

The reasons and mechanisms leading to the increased ovulation rate and physiologic characteristics of FL1 have never been examined and remain unclear. This study aims to characterize the Dummerstorf high-fertility mouse line FL1 on endocrine and molecular levels to draw a global picture of how the FL1 phenotype of high-fertility is achieved. We analyzed parameters of the HPG axis and performed a holistic gene expression approach in the ovary to determine whether the increased ovulation rate of FL1 mice is due to local mechanisms in the ovary or if it is rather a consequence of systemic alterations in the HPG axis.

## Materials and methods

### The Dummerstorf mouse lines ctrl and FL1

Both mouse lines are descendants of the same outbred mouse line, which was generated by cross-breeding of four different inbred and four outbred mouse lines (Schüler 1985). The ctrl line did not undergo any selection process. However, a rotational mating scheme with 125–200 breeding pairs per generation has been used to minimize the degree of kinship. The FL1 line was created by long-term selection. They were selected for a combination of the number of pubs per litter and the total birth weight of the litters at birth of primiparous females (Dummerstorf fecundity index = 1.6× litter size + litter weight) for 162 generations, followed by Best Linear Unibased Prediction breeding value estimation, focusing only on higher offspring per female. A total of 60–100 mating pairs were used per generation (Schüler 1985, Spitschak *et al.* 2007). Hence, the FL1 mouse line is much more heterogeneous than the classical inbreed or transgenic mouse lines. Table 1 shows the body mass, litter size, total litter weight, and weight per pub of FL1 mice and ctrl mice of the generation used in this study.

**Table 1:** Body mass, total litter size at birth, litter weight at birth, and mean birth weight per pub of the mouse generations used in this study.

	Generation number	Body mass at day 63 (g)		Total litter size at birth		Litter weight at birth (g)		Mean birth weight per pub (g)	
		*n*	Mean ± s.d.	*n*	Mean ± s.d.	*n*	Mean ± s.d.	*n*	Mean ± s.d.
*ctrl*	192	310	31.0 ± 2.9	119	11.3 ± 2.9	119	19.8 ± 4.7	119	1.79 ± 0.2
*FL1*	191	114	35.2 ± 3.2	55	21.0 ± 4.0	55	36.9 ± 5.0	55	1.79 ± 0.2

### Animals and housing

All procedures were performed following national and international guidelines and approved by the institutional board (Animal Protection Board from the Research Institute for Farm Animal Biology). Female mice, bred at the Research Institute for Farm Animal Biology (FBN), Dummerstorf, Germany, were housed in groups of three animals per cage. A commercial breeding diet for rodents and water was provided *ad libitum,* and illumination of animal facilities was between 06:00 h and 18:00 h. In addition, a male mouse was kept for acoustic, visual, and olfactory stimulus in a separate cage.

### Determination of the estrous cycle

The murine estrous cycle takes 4–5 days and is generally divided into four stages: proestrus, estrus, metestrus, and diestrus. During the estrous cycle, female mice are subject to major changes, which occur at different levels. Cycle-related regulation of gene expression, hormonal alterations in the HPG axis, or anatomical changes in several tissues may affect results of research. Therefore, samples were taken in estrus and diestrus. Vaginal cytology is the most accurate method to detect the stages of the estrous cycle in mice and was used in this study. There are various references on the implementation and evaluation of vaginal cytology in mice (Byers *et al.* 2012, Cora *et al.* 2015). At the age of 65 ± 3 days, vaginal smears were performed daily at 9.00 h for 12 days. Therefore, a 10 µL drop of PBS (Roti®-CELL PBS, Carl Roth GmbH + Co. KG, Karlsruhe, Germany) was placed at the opening of the vaginal canal and was pipetted up and down three to four times, without penetrating or injuring the vagina. Then the recovered PBS drop, containing flushed cells of the vaginal mucosa, was placed on a glass slide and was immediately evaluated under a light microscope (Nikon ECLIPSE TE2000-S, 10× magnification). In this study, we analyzed samples taken in diestrus (*n* ctrl = 10, *n* FL1 = 10) and estrus (*n* ctrl = 10, *n* FL1 = 10). To keep the time point of sampling within estrus or diestrus as constant as possible, samples were taken when exclusively cornified epithelial cells (no other cells) appeared in vaginal smears in estrus, respectively, only leucocytes in diestrus.

### Sample procedure

Animals at the age of 77 ± 5 days were euthanized by CO_2_ inhalation after the stage of the estrous cycle had been determined. Samples were taken at the same daytime. After determination of death, the thorax was opened. The *Vena cava caudalis* was cut, and blood was collected out of the thorax. Blood samples were left for 2 h at room temperature. The resulting blood clot was removed, and the supernatant was centrifuged (4°C, 2000 ***g***, 10 min). Both ovaries were extracted and cleaned from fat tissue. *Tunica albuginea* and oviduct were removed. In case of estrus, the oviduct was opened and the cumulus oocyte complex was taken out. The oocytes were isolated and counted. Each ovary was weighed. In addition, the uterus was cleaned from fat tissue and weighed. The weight of the reproductive organs is shown in additional file 1. Pituitary and hypothalamus were extracted. All samples were immediately snap-frozen and stored at −70°C.

### qPCR

Hypothalamus samples of 10 animals per line (estrus *n*  = 5; diestrus *n*  = 5) and pituitary samples of 18 animals per line (estrus *n*  = 9; diestrus *n*  = 9) were used for qPCR analysis of *Gnrh* (hypothalamus), *Fsh,* and *Lh* (pituitary).

Hypothalamus and pituitary were pulverized in liquid nitrogen. RNA was extracted with an RNeasy Plus Micro Kit (Qiagen) and reverse transcribed using an iScript cDNA Synthesis kit (Bio-Rad) according to the manufacturers’ protocol. Primers were designed using Primer-BLAST and purchased from TIB Molbiol (Berlin, Germany). Each sample was analyzed using 4 µL primer mix, 5 µL iQ SYBR Green Supermix (Bio-Rad), and 1 µL of cDNA reaction solution and was loaded onto a 96-well plate and amplified by real-time PCR (iCycler, Bio-Rad). In addition, the PCR products of *Gnrh, Fsh, and Lh* were cleaned using the NucleoSpin® Gel & PCR Clean up kit (Macherey-Nagel, Düren, Germany) and used as standard curve (100–0.1 fg/µL) on which the total mRNA abundance in the samples was determined. The results of the mRNA abundance were calculated relative to a combination of the reference genes *Rps18*, *36b4*, *Gadph*, and *B2m*. The sequences of the primers and reference genes are shown Supplementary file 1 (see section on supplementary materials given at the end of this article).

### Hormonal analysis

Serum samples of 18 animals per line were used for hormonal analysis (estrus *n*  = 9; diestrus *n*  = 9). For the measurement of FSH and LH, undiluted serum samples were assayed (repeated measurement) using an MPTMAG assay kit (Merck Millipore) on a Luminex LX200 system according to the manufacturer’s instructions. According to the manufacturer, the sensitivity of the assay is 9.5 pg/mL for FSH and 4.9 pg/mL for LH. For both FSH and LH, the intraassay variation is below 15% and interassay variation is below 20% as given by the manufacturer.

Serum concentrations of progesterone (P4) were determined in duplicate using a competitive single-antibody ^3^H-RIA with [1,2,6,7-3H] progesterone tracer (Hartmann Analytic, Germany), as previously described (Brüssow *et al.* 2011, Michaelis *et al.* 2017). Briefly, an ether extraction was performed. Radioactivity was counted using a liquid scintillation counter with an integrated RIA program (TriCarb 2900 TR; Perkin-Elmer). This assay has a sensitivity of 7 pg/mL. The intraassay variation was 7.6%, and the interassay variation was 9.8%.

### Statistical analysis

Statistical analysis of the results of the hormonal analysis was performed with the program IBM SPSS Statistics, using the Mann–Whitney U test. All data points have been included in the analysis, and no outlier data points have been omitted.

### Transcriptome analysis

Ovary samples of 10 animals per line (estrus *n*  = 5; diestrus *n*  = 5) were used for the transcriptome analysis. RNA was extracted using the RNeasy Plus Micro kit (Qiagen). Libraries were prepared with the QuantSeq 3’ mRNA-Seq Fw. Library Prep kit (Lexogen) and sequenced to 10 M raw reads on an Illumina HiSeq 2500 machine, following the manufacturer’s protocols. Data were pre-processed and analyzed using the options recommended by the manufacturer. Trimmed sequences were aligned with STAR aligner 2.6.0a (Dobin *et al.* 2013) against the GRCm38 (release 95) mouse genome retrieved from the Ensembl database via biomaRt (Smedley *et al.* 2009) tool. In average, 7.28 M reads per sample were uniquely assigned. In total, 55,754 genes were annotated, but not all of these genes were expressed, and 18,500 annotated transcripts had at least 1 read. The trimmed read length was 51 ntd.

Raw sequence data and raw counts were deposited at the NCBI GEO repository, entry GSE174082. Differential analysis was performed with the DESeq2-package v1.14.1 (Love *et al.* 2014) in R 4.0.0.

### Re-evaluation of the transcriptome analysis with qPCR

A total of 14 differentially expressed genes were picked out and analyzed using qPCR to re-evaluate the results of the transcriptome analysis. For this validation experiment, 20 animals per line (estrus *n*  = 10; diestrus *n*  = 10) were used. The RNA was extracted and reverse transcribed like described above. Each sample was analyzed in duplicates using 4 µL primer mix, 5 µL iQ SYBR Green Supermix (Bio-Rad) and 1 µL of cDNA reaction solution and was loaded onto a 96-well plate and amplified by real-time PCR (iCycler, Biorad, München, Germany). The results of the mRNA abundance are calculated relative to a combination of reference genes *RPS18*, *36B4,* and *B2m*. Relative gene expression was calculated using the relative expression software tool (Pfaffl 2001, Pfaffl *et al.* 2002). The sequences of the primers and reference genes are shown in Supplementary file 1.

## Results

### Ovulation rate and phenotypic description

**Figure 1:**
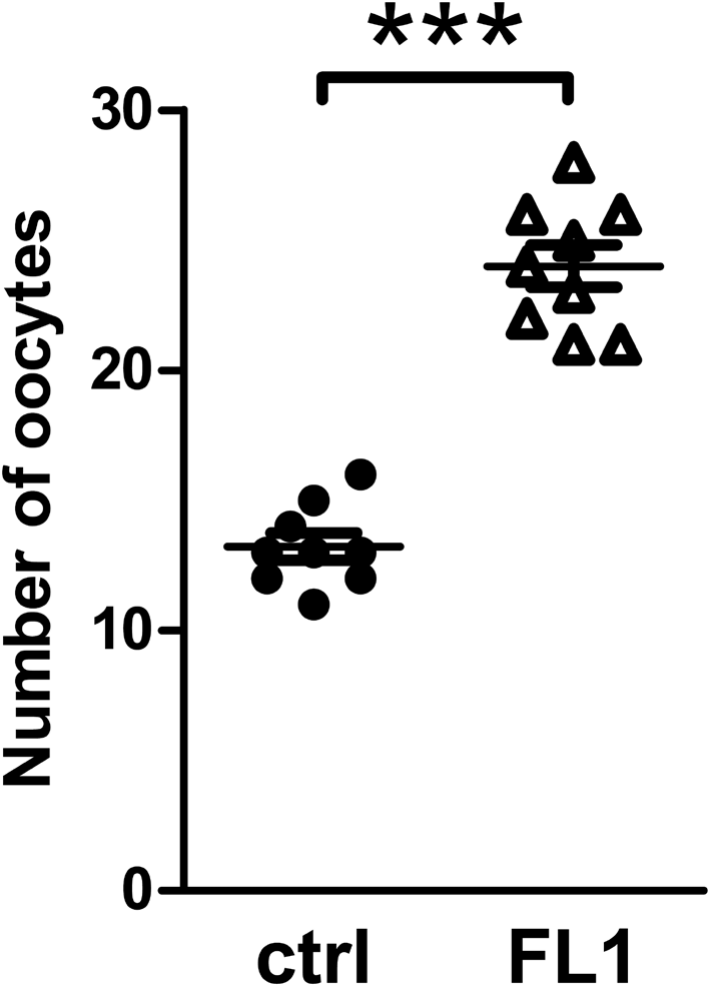
Number of ovulated oocytes in ctrl mice and FL1 mice. The oocytes were isolated out of the cumulus in the oviduct in cycle controlled animals in estrus. Data are expressed as mean ± s.e.m., ***P  < 0.001 (Mann–Whitney U test).

**Figure 2:**
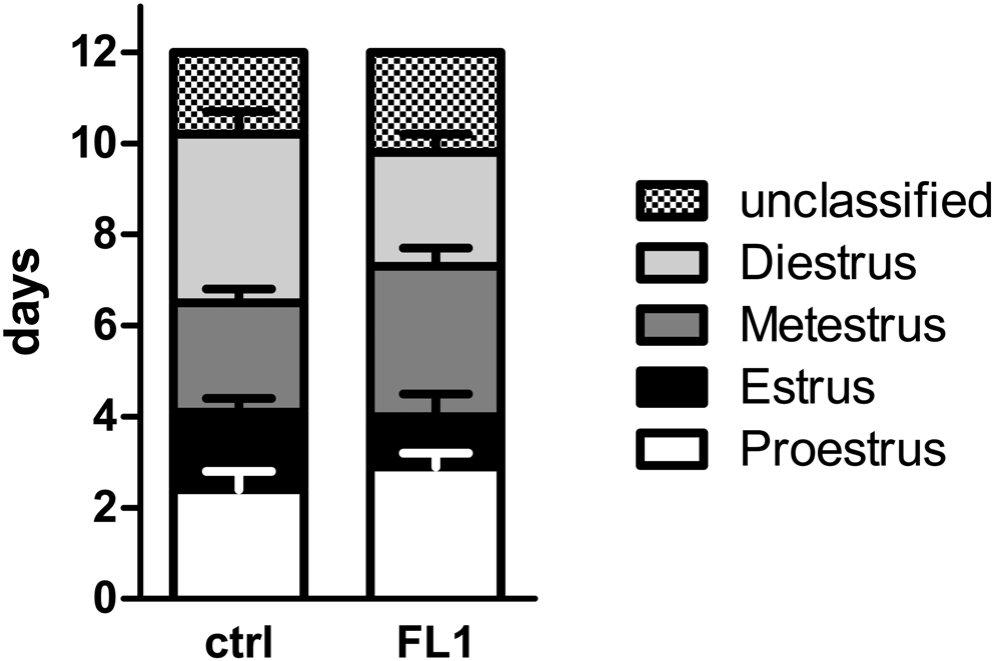
Distribution of the total time of the stages of estrous cycle in ctrl mice and FL1 mice over a time period of 12 days. Data are expressed as means + s.e.m. of the timeframes in the different stages of estrous cycle. The distribution of the stages of estrous cycle was not significantly different between ctrl mice (*n*  = 10) and FL1 mice (*n*  =10) (Mann–Whitney U test).

During the selection process, FL1 doubled the number of offspring per litter. Therefore, higher numbers of ovulated oocytes in the oviduct are manifested. FL1 females ovulate nearly twice as many oocytes as ctrl females (Fig. 1). To further characterize FL1 female mice, the weights of the reproductive organs were analyzed. We found no significant difference in the weight of the ovaries or the uterus between lines (Supplementary file 2). The estrous cycle was monitored over a 12 days observation period (Fig. 2). The distribution of the stages of estrous cycle was not significantly different between ctrl and FL1 mice during the observation period of 12 days (100 %). Estrus was determined in ctrl mice in 1.7 ± 0.3 days (14%) compared to 1.1 ± 0.5 days in FL1 mice (9%). Metestrus was determined in 2.4 ± 0.3 days (20%) in ctrl mice and 3.3 ± 0.4 days (34%) in FL1 mice. Ctrl mice remain 3.7 ± 0.5 days and FL1 mice 2.5 ± 0.4 days in diestrus, which corresponds to 31% (ctrl) and 22% (FL1). Finally, times in proestrus differ from 2.4 ± 0.4 days (20%) in ctrl mice to 2.9 ± 0.3 days (16%) in FL1.

### Expression of *Gnrh* in the hypothalamus

**Figure 3:**
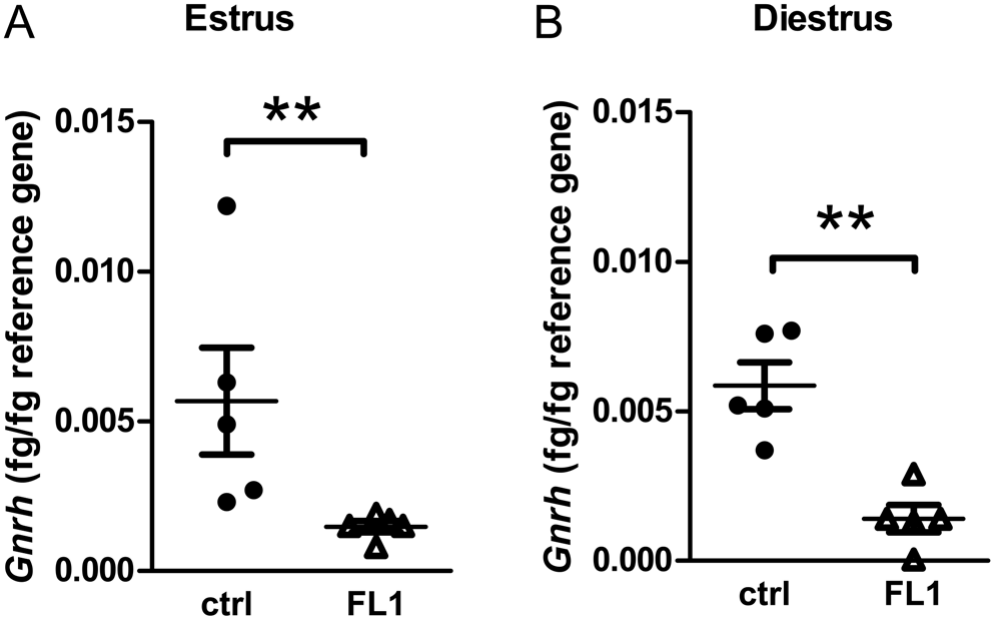
mRNA content of *Gnrh* (fg/fg reference gene) in ctrl mice and FL1 mice in estrus (A) and diestrus (B). Data are expressed as mean ± s.e.m., ** *P*  < 0.01 (Mann–Whitney U test).

To address the question of why FL1 females ovulate twice as many oocytes compared to ctrl females, hormones of the HPG axis were analyzed. The mRNA content of *Gnrh* in estrus was decreased by more than 70% in FL1 mice compared to ctrl mice. Accordingly, the mRNA content of *Gnrh* in diestrus was significantly lower in FL1 than in ctrl (Fig. 3).

### Expression and serum concentrations of pituitary hormones

**Figure 4:**
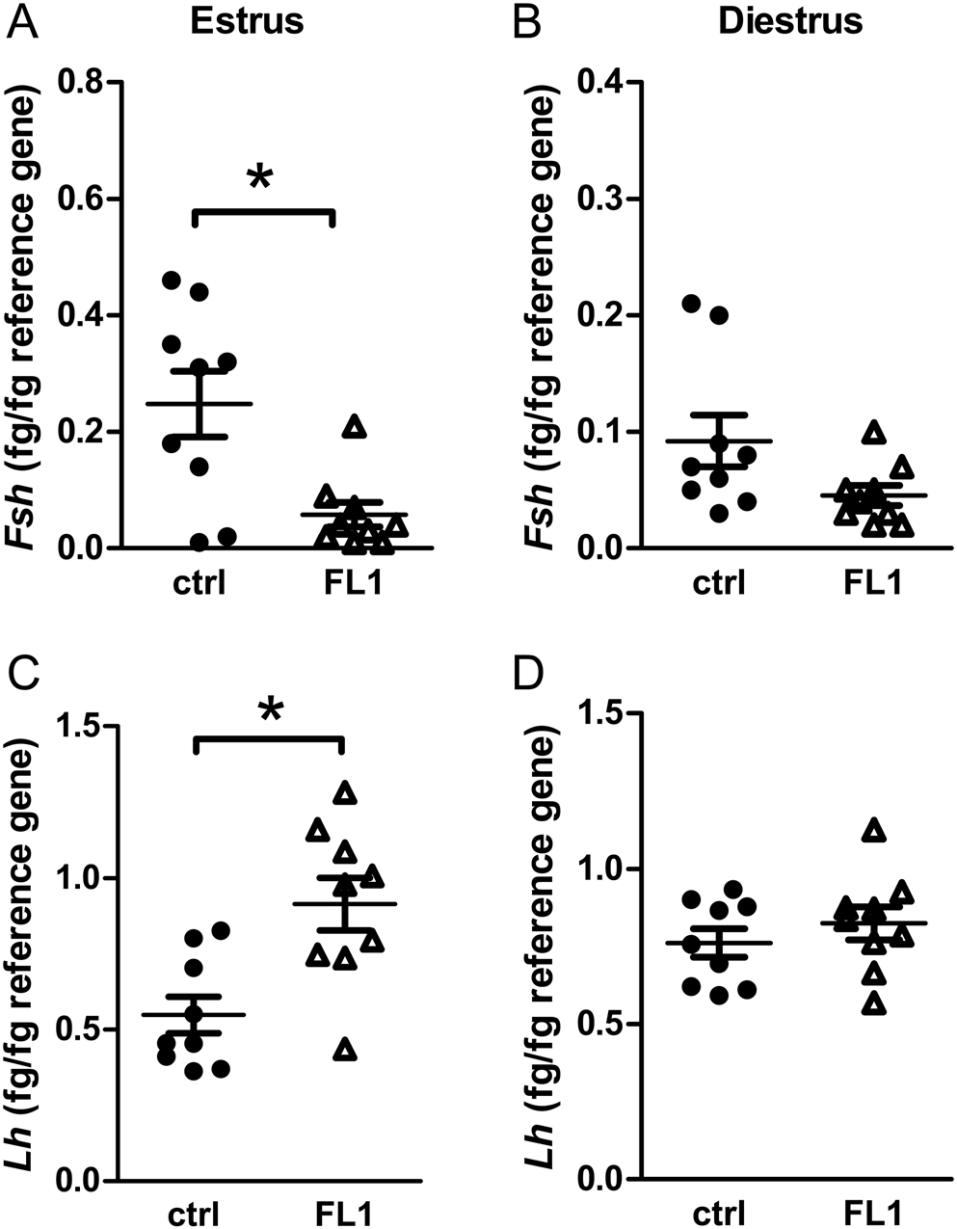
mRNA content of the pituitary hormones *Fsh* and *Lh* in ctrl mice and FL1 mice in estrus and diestrus. *Fsh* mRNA content (fg/fg reference gene) in estrus (A) and diestrus (B). *Lh* mRNA content (fg/fg reference gene) in estrus (C) and diestrus (D). Data are expressed as mean ± s.e.m., * *P*  < 0.05 (Mann–Whitney U test).

**Figure 5:**
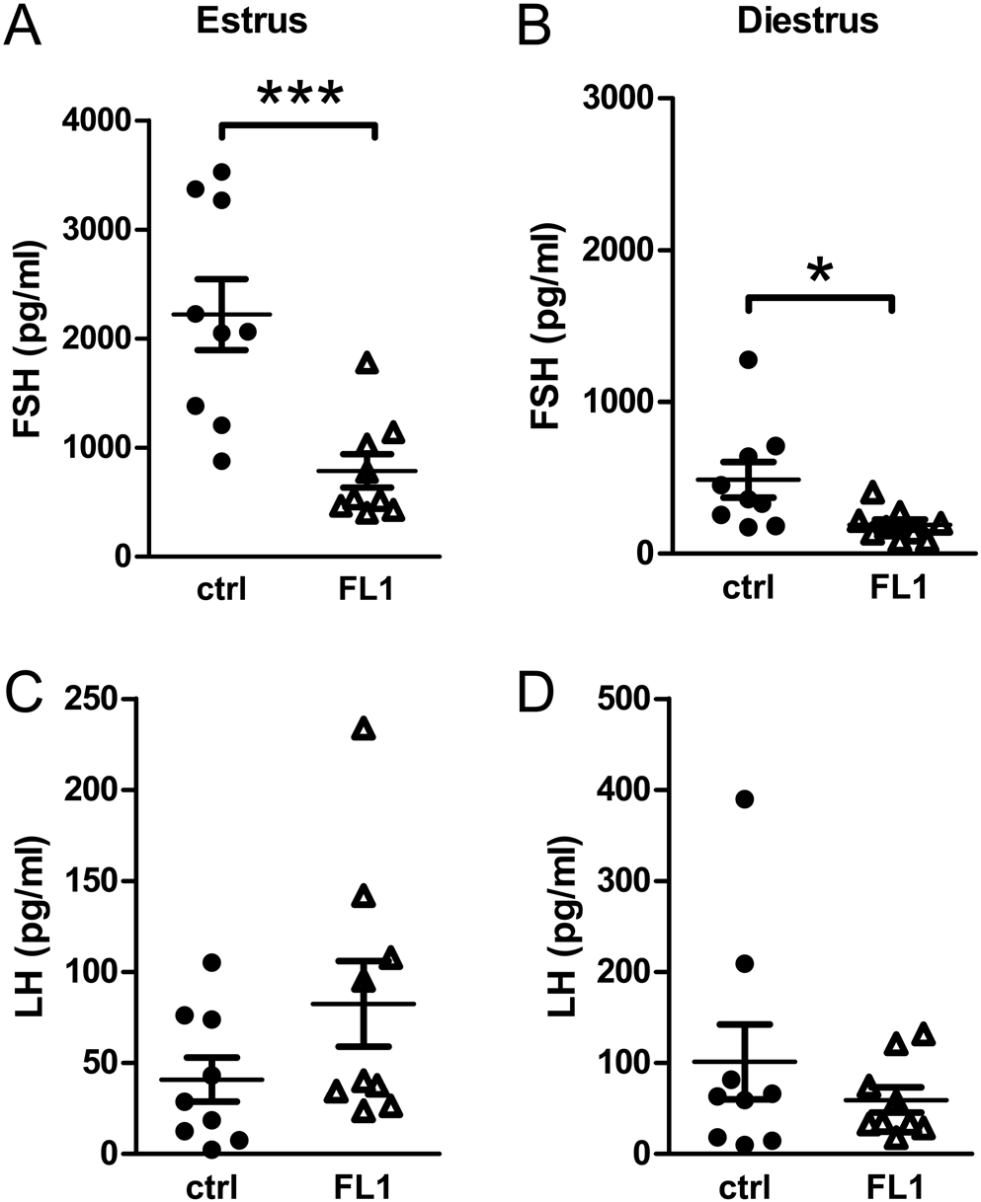
Serum concentration of the pituitary hormones FSH and LH. Serum concentration of FSH (pg/mL) in ctrl mice and FL1 mice in estrus (A) and diestrus (B). Serum concentration of LH (pg/mL) in ctrl mice and FL1 mice in estrus (C) and diestrus (D). Data are expressed as mean ± s.e.m., * *P*  < 0.05, *** *P*  < 0.001 (Mann–Whitney U test).

FSH was analyzed on both transcriptional (Fig. 4A andB) and protein levels (Fig. 5A and B). Overall, we found a significant reduction in FSH protein and transcript levels in FL1 mice compared to ctrl mice. FSH concentrations were significantly lower in FL1 (788.4 ± 152.1/mL) compared to ctrl (2220.2 ± 327.4 pg/mL) in estrus (*P*  = 0.0008). In diestrus, FSH concentrations in serum were also lower in FL1 (191.9 ± 33.7 pg/mL) compared to ctrl (488.1 ± 116.6 pg/mL; *P*  = 0.014). At the mRNA level, we also detected lower mRNA content in estrus and diestrus of FL1 compared to ctrl.

In contrast, the mRNA level of* Lh* was increased in estrus of FL1 mice (Fig. 4C). Although statistically not significant, LH concentrations tended to be twofold increased in FL1 mice in estrus (Fig. 5C). In diestrus, LH was not significantly different in FL1 and ctrl on both mRNA (Fig. 4D) and protein (Fig. 5D) levels.

### Concentration of sex steroids

**Figure 6:**
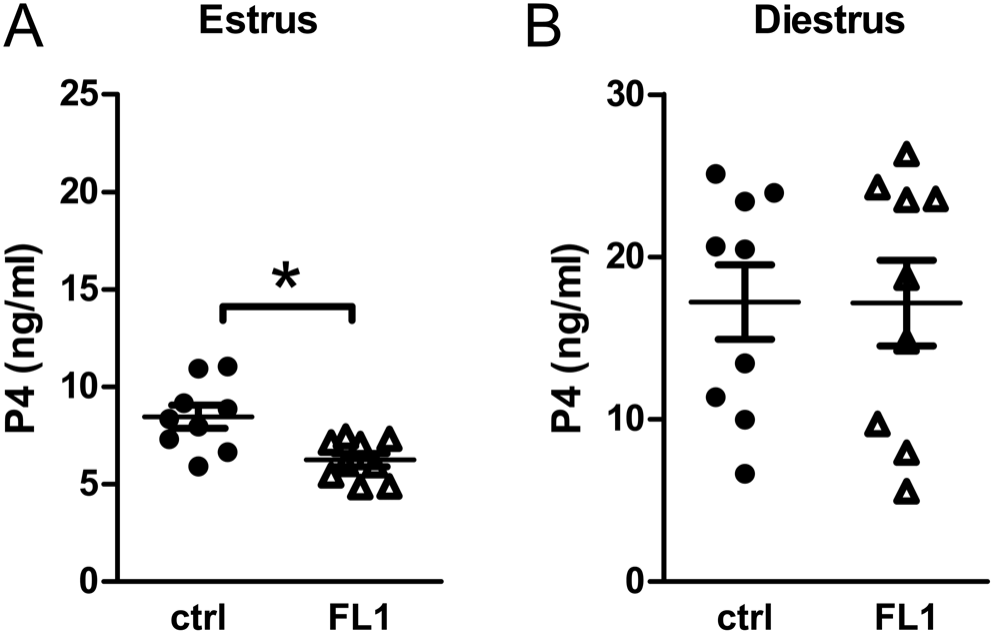
Serum concentration of P4 (ng/mL) in ctrl mice and FL1 mice in estrus (A) and diestrus (B). Data are expressed as mean ± s.e.m.,* *P*  < 0.05 (Mann–Whitney U test).

P4 levels were analyzed in the serum (Fig. 6A and B). In estrus of FL1, we found significantly lower P4 serum concentrations (6.3 ± 0.1/mL) compared to ctrl (8.5 ± 0.6 ng/mL). We found no significant difference in P4 concentration between FL1 (17.2 ± 2.6 ng/mL) and ctrl (17.2 ± 2.3 ng/mL) in diestrus.

### Transcriptome analysis and re-evaluation with qPCR

**Figure 7:**
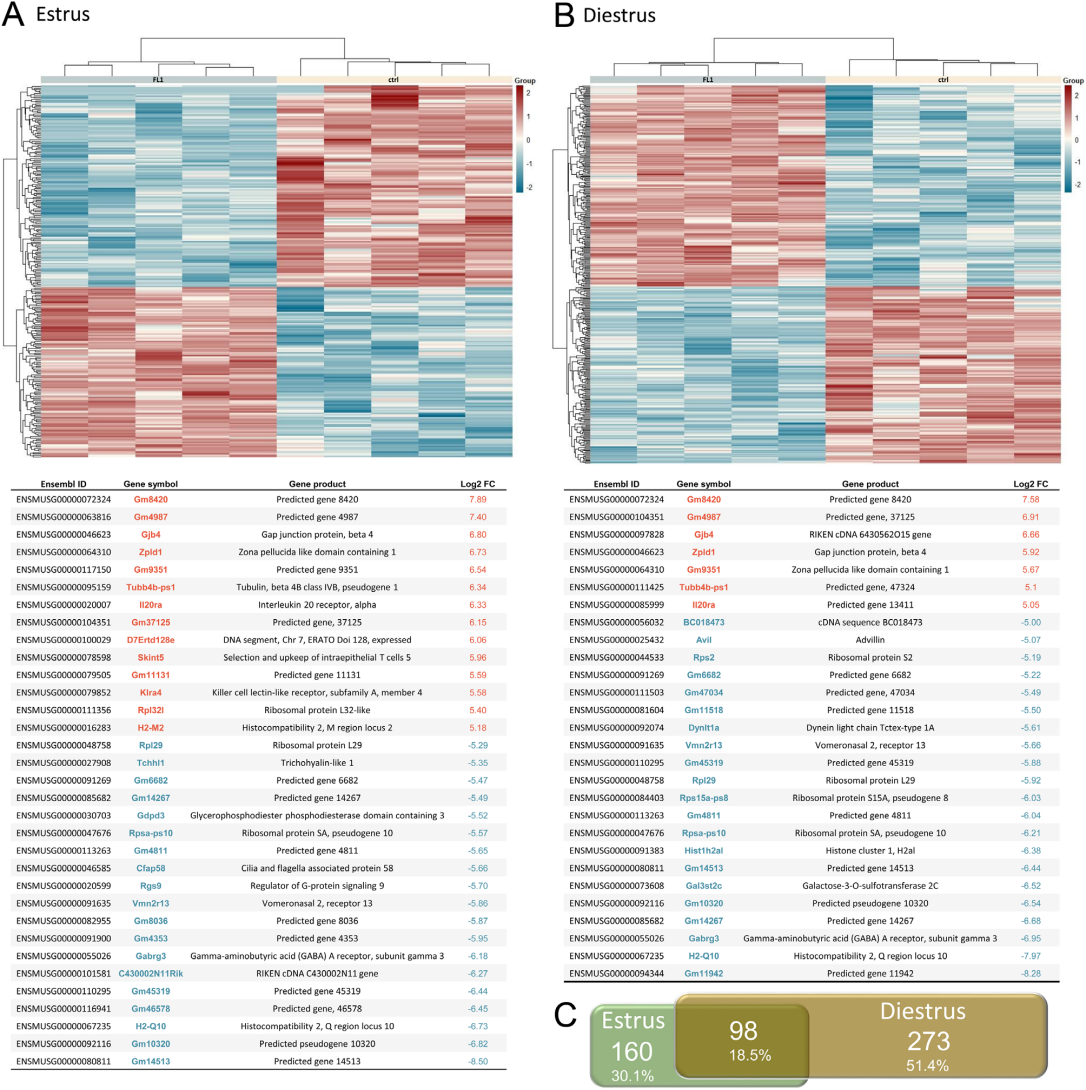
Hierarchical clustering dendrograms of differentially expressed genes (log2FC) in (A) estrus and in (B) diestrus of FL1 and ctrl mice. High- and low-expression intensities are represented by red and blue color, respectively (see scale on the right margin). Features with higher and lower transcripts levels (with log2FC > 5; *P*  < 0.01) in FL1 compared to ctrl mice are listed below the heatmaps. (C) A Venn diagram illustrates the absolute number and the percentage of DE features in the estrus and diestrus of FL1 mice relative to the expression values obtained for ctrl mice. The overlap depicts the proportion of DE genes shared by both mouse lines. The diagram was calculated based on the official gene symbols. A full color version of this figure is available at https://doi.org/10.1530/JME-22-0012.

**Figure 8:**
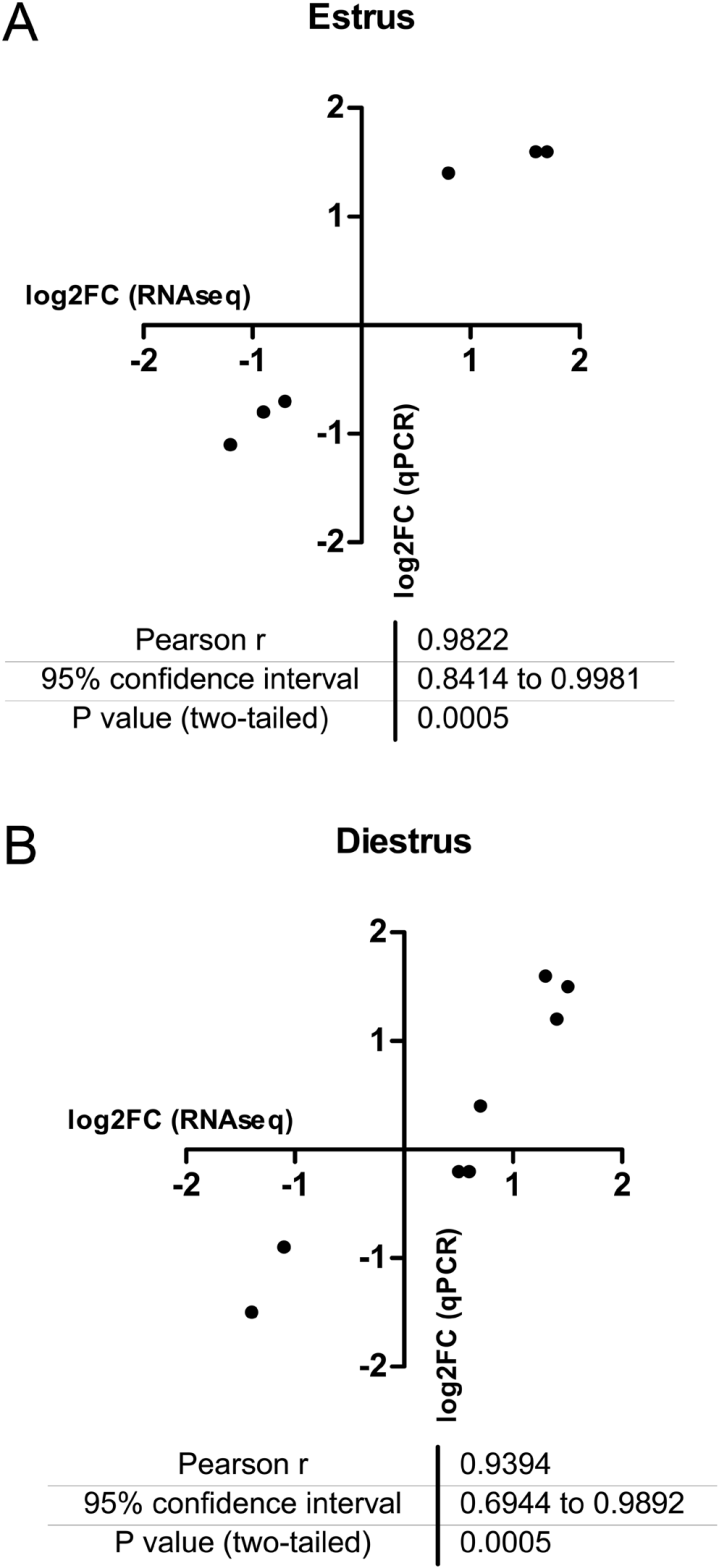
Concordance and correlation of the results of the transcriptome analysis and the qPCR. The log2FC is for mRNAseq and qPCR for selected genes is shown for samples in estrus (A) and in diestrus (B). A full color version of this figure is available at https://doi.org/10.1530/JME-22-0012.

A transcriptomic analysis of estrus and diestrus from ctrl and FL1 mice revealed line-specific expression patterns (Fig. 7A and B). Compared with ctrl mice, 258 genes were differentially regulated (84 stronger and 174 weaker expressed genes with *q* ≤ 0.05) in estrus, while 371 genes were differentially regulated (174 stronger and 197 weaker expressed genes with *q* ≤0.05) in diestrus of FL1 mice. Both lists of differentially expressed genes share 98 common genes (Fig. 7C), and contain key and well established genes involved in female reproductive performance (Tables 2 and 3). A selection of genes associated with different traits of female fertility, which are not discussed in detail in this article, is provided in Supplementary file 3. qPCR on selected genes was used to re-evaluate the transcriptomic data. The correlation between log2FC of the transcriptome sequencing and the qPCR analysis in estrus (Fig. 8A) and in diestrus (Fig. 8B) ranged between 0.93 and 0.98.

**Table 2:** List of genes associated with reproductive traits in estrus.

Gene	ΔLog2FC	ΔLog2FC qPCR	Keyword
Genes associated with the reproductive lifespan			
*Per2*	−1.5^a^	−1.4^a^	Absent estrous cycle, decreased litter size, reproductive lifespan (Pilorz & Steinlechner 2008)
*Kl*	1.6^a^	1.6^b^	Reproductive lifespan (Kuro-o *et al.* 1997)
*Tex14*	2.1^c^		Reproductive lifespan (Greenbaum *et al.* 2009)
Genes associated with folliculogenesis			
*Igfbp2*	−0.9^b^	−0.8^b^	Follicular development (Spitschak & Hoeflich 2018); follicular atresia (Wandji *et al.* 1998)
*Esr1*	−0.7^a^	−0.7^b^	Granulosa cell proliferation (Dupont *et al.* 2000); folliculogenesis (Schomberg *et al.* 1999, Couse & Korach 1999); alteration of LH and FSH levels (Adlanmerini *et al.* 2014); ovulation rate (Jakacka *et al.* 2002)

The ΔLog2FC is calculated based on the result of the transcriptome analysis (^a^*q* < 0.05, ^b^*q* < 0.01, ^c^*q* < 0.001, *n*  = 5). Additionally, the ΔLog2FC based on the results of the qPCR validation experiment is shown (*n*  = 10).

**Table 3:** List of genes associated with reproductive traits in diestrus.

Gene	ΔLog2FC	ΔLog2FC qPCR	Keyword
Genes associated with the reproductive lifespan			
*Rora*	0.7^a^	0.7^a^	Estrous cycle, reproductive life span (Guastavino & Larsson 1992); number of mature oocytes (Guastavino *et al.* 2005)
Genes associated with folliculogenesis			
*Kit*	−1.4^a^	−1.5^b^	Folliculogenesis (Pangas & Rajkovic 2015, Reynaud *et al.* 2001); litter size (Geissler *et al.* 1981); ovarian follicle number (Geissler *et al.* 1981)
*Nos2*	−0.9^b^		Litter size (Burnett *et al.* 2002); ovulation rate, ovarian morphology, steroidogenesis (Jablonka-Shariff *et al.* 1999), marker of follicular atresia (Nath & Maitra 2019)
*Foxl2*	0.5^b^	−0.2	Folliculogenesis and granulosa cell differentiation (Schmidt *et al.* 2004, Nicol *et al.* 2020)
*Smad1*	0.8^b^	1.4^a^	Primordial germ cells (Tremblay *et al.* 2001); folliculogenesis (Pangas 2012)
*Lhcgr*	1.4^a^	1.2^b^	Folliculogenesis, formation of corpus luteum (Zhang *et al.* 2001, Lei *et al.* 2001, Hunzicker-Dunn & Mayo 2006); levels of FSH, LH, P_4_ and E_2_ (Lei *et al.* 2001, Hunzicker-Dunn & Mayo 2006)
*Cxcr4*	1.5^c^	1.5^a^	Primordial germ cell migration (Molyneaux *et al.* 2003); granulosa cell survival (Molyneaux *et al.* 2003, Kryczek *et al.* 2005)

The ΔLog2FC is calculated based on the result of the transcriptome analysis (^a^ *q* < 0.05, ^b^ *q* < 0.01, ^c^ *q* < 0.001, *n*  = 5). Additionally, the ΔLog2FC based on the results of the qPCR validation experiment is shown (*n*  = 10).

## Discussion

The Dummerstorf high-fertility mouse line FL1 almost doubled the litter size during its selection process to 21.0 pups/litter compared to 11.3 pups/litter in the unselected control line. The aim of this study was to get insights into how the improved fertility phenotype is warranted on endocrine and molecular levels.

In previous work, it has already been described that FL1 mice ovulate more oocytes than the corresponding controls (Spitschak *et al.* 2007). This was verified in our recent analysis. In average ctrl mice ovulated 13.2 oocytes per cycle, whereas FL1 ovulated 22.4 oocytes per cycle.

We also analyzed several phenotypic parameters to document how the phenotype shifted due to the long-time selection process. We did not observe any significant differences in the weight of reproductive organs between FL1 and ctrl. The analysis of the estrous cycle of FL1 and ctrl indicates slight but not significant differences concerning the length of various cycle stages.

In order to address endocrine and molecular reasons for the improved ovulation phenotype, we measured hormones of the HPG axis. Since it is widely accepted that FSH or substances such as pregnant mare serum gonadotropin, which simulate the effects of FSH, are used to improve ovulation rates, we expected higher FSH concentrations in FL1 compared to ctrl. Surprisingly, this was not the case. We measured decreased *Fsh* gene expression in the pituitary and decreased hormone concentrations in serum, both in estrus and in diestrus. The dampened FSH concentrations were accompanied by decreased hypothalamic *Gnrh* transcript levels in estrus and diestrus. Thus, we have to sharpen our view on FSH. Obviously, external supplementation of FSH helps to improve ovulation rates, and in cases where there is a lack of FSH due to mutations in the coding sequence, ovulation can be induced by administration of exogenous FSH (Matthews *et al.* 1993). Overexpression of *Fsh* in transgenic mice initially leads to higher ovulation rates and higher litter sizes. However, these mice suffer from accelerated reproduction failure and premature infertility (McTavish *et al.* 2007). However, this phenotype is not seen in FL1 mice. Hence, high FSH concentrations might increase ovulation rates when given once or in cases of deficient FSH, whereas long-term overexpression rather seems to lead to decreased lifetime fecundity. However, this female phenotype differs from the phenotype of FL1, where we detected high ovulation rates (accomplished by large litter sizes) plus a superior lifetime fecundity (Langhammer *et al.* 2021). It is conceivable that decreased FSH concentrations might be compensated by an increased sensitivity of the FSH receptor. However, activating mutants are very rare, while several naturally occurring mutations which inactivate the FSH receptor gene have been described. Peltoketo *et al.* (2010) created knock-in mice with significantly increased basal activity of the FSH receptor and showed that this activating mutation results in enhanced granulosa cell proliferation, loss of small follicles, and the development of hemorrhagic cysts; therefore, most of the mice were infertile (Peltoketo *et al.* 2010). In view of this fact, also this phenotype differs from the FL1.

We observed significantly higher level of *Lh* transcripts in pituitary in FL1 mice compared to ctrl mice in estrus but not in diestrus. Serum concentrations of LH in estrus of FL1 were also slightly higher, although statistically not significant. Although the role of LH in the murine reproductive cycle and its effects on follicles in the antral stage is well established, quite less is known about the function of LH in folliculogenesis in the preantral stage. *In vitro* experiments by Wu *et al.* indicate that LH and FSH are required for the development of small preantral follicles to antral stage, while bigger preantral follicles only needed the addition of FSH in the medium to reach the antral stage (Wu *et al.* 2000). This suggests that LH mediates several important functions in the development of small preantral follicles. Although not experimentally tested, increased LH levels in FL1 might promote the development of small preantral follicles. Moreover, addition of LH as an exogenous hormone to ovarian tissue results in increased numbers of healthy follicles and recovery time of the estrous cycle after pregnancy (Zheng *et al.* 2020). Obviously, the increased LH formation in FL1 has various promoting effects on different processes in folliculogenesis and reproductive performance.

We found significantly lower P4 concentrations in estrus in FL1, and no difference between ctrl and FL1 in the serum concentrations of P4 in diestrus. A dose-dependent effect of P4 on follicular growth has been shown in studies using cultured ovaries. Addition of a medium with a low concentration of P4 resulted in enhanced growth of follicles from primordial to primary stage. In contrast, high P4 in the medium suppressed the growth of secondary follicles (Komatsu & Masubuchi 2017). Kezele and Skinner (2002) observed the effects of different P4 concentrations on primordial follicles in newborn rat ovaries. Also, this study revealed that high P4 concentrations inhibited the primordial to primary follicle transition. Furthermore, high P4 concentrations resulted in significantly decreased primordial follicle assembly *in vivo* and *in vitro*, which suggests a role of maternal P4 in the control of fetal primordial follicle assembly and early follicular development (Kezele & Skinner 2003). Thus, lower P4 concentrations have the potential to increase the primordial follicle activation in FL1. It is also conceivable that decreased maternal steroid concentrations promote the early follicular development of newborn pubs.

Our results of the measurements of hormones suggest that the increased ovulation rate of FL1 is not only due to systemic alterations in the HPG axis. Although altered levels of LH and P4 might promote follicular development, it furthermore seems to be regulated by local mechanisms in the ovary.

Considering that only less than 1% of the initially formed primordial follicles succeed in entering maturation and ovulation (Bjersing 1982), it is conceivable that increased primordial activation can be a mechanism that is jointly responsible for increased reproductive performance. Although several genes are known, which are involved in the activation of primordial follicles, the mechanisms and pathways are widely unclear (Pangas & Rajkovic 2015). One of these genes is *Foxl2*, which is stronger expressed in the diestrus of FL1 compared to ctrl mice. The formation of primordial follicles is not impaired in *Foxl2* *^−/−^* mice during the first three postnatal days, but they never contain more than one granulosa cell layer, undergo atresia, and therefore, fail in further folliculogenesis (Schmidt *et al.* 2004). Interestingly, overexpression of *Foxl2* results in formation of POFs as a consequence of defects in the nest breakdown during ovarian development (Nicol *et al.* 2020). As already described above, the occurrence of POFs is characteristic for FL1 (Alm *et al.* 2010). However, the formation of POFs occurs in the time period between the breakdown of the germ cell clusters and the development of the primordial follicles and not in the adult ovary, and therefore, the results of this study cannot be used to explain mechanisms that occur during ovarian development. The role of the the POFs in the adult ovary and how they contribute to the increased reproductive performance of FL1 females is not clear. Although the occurrence of POFs in primary, secondary, and antral follicles, as well as the fact that POFs are able to reach ovulatory diameter, suggests that they are able to ovulate (Alm *et al.* 2010), it is not known if multiple oocytes originated from POFs can be fertilized and develop into living offspring. Spitschak *et al.* (2007) analyzed the numbers of released oocytes, corpora lutea, and living fetuses in FL1 mice. Both, the number of released oocytes and the number of coprpora lutea were significantly higher in FL1 compared to ctrl mice. However, not all released oocytes developed into living offspring. While the release of oocytes was significantly higher than the number of corpora lutea in FL1 mice, the number of corpora lutea was in accordance with the number of living fetuses (Spitschak *et al.* 2007). Thus, the number of offspring seems to be determined by the number of corpora lutea. Since one follicle develops into one corpus luteum after ovulation, it is not likely that every oocyte that is released out of a POF is able to develop into living offspring.

*Kit* is a potential regulator of primordial follicle activation, as it activates the phosphatidylinositol 3-kinase-protein kinase B (PI3K-AKT) pathway. *Kit* has been found differentially regulated in the diestrus of FL1 vs ctrl. Several reports on mouse models with mutations in the *Kit* loci or targeted disruption of this gene discuss the functions of *Kit* in folliculogenesis (Reynaud *et al.* 2001, Pangas & Rajkovic 2015). Aside from the primordial follicle activation, the PI3K-AKT pathway regulates the growth and survival of cells in various cell types (Pangas & Rajkovic 2015, Chu *et al.* 2018, Kim & Kurita 2018) and therefore has the potential not only to regulate the primordial activation but might also be involved in the modulation of follicular growth and survival during folliculogenesis in FL1.

*Esr1* is well known to be involved in the process of folliculogenesis (Couse & Korach 1999, Schomberg *et al.* 1999). FL1 mice have lower *Esr1* levels in estrus (Table 2). Especially, the primordial follicles seem to be controlled by *Esr1* (Arao *et al*. 2011, Adlanmerini *et al*. 2014). Furthermore, it plays a crucial role in granulosa cell proliferation (Dupont *et al.* 2000) and affects the concentrations of LH and FSH (Adlanmerini *et al.* 2014). It has also been shown that litter size and ovulation rate are influenced by *Esr1* (Jakacka *et al.* 2002).

*Igfbp2*, which is on lower levels in the estrus of FL1, is involved in follicular growth, differentiation, and degeneration (Spitschak & Hoeflich 2018). Especially the potential function in follicular atresia is remarkable. Wandji *et al.* (1998) observed that the levels of *Igfbp2* mRNA are associated with follicular atresia (Wandji *et al.* 1998). The results of this study lead to the assumption that lower amounts of *Igfbp2* transcript results in decreased atresia of the follicles and thus a higher ovulation rate in FL1.

*Cxcr4,* which is more abundant in the diestrus of FL1 compared to ctrl mice, is an example for a gene associated with follicular survival. The ligand/receptor interaction of Cxcr4 and its ligand C-X-C motiv-chemokine 12 (*Cxcl12*) is involved in the chemotaxis of several cell types and plays a role in granulosa cell survival in the pre- and periovulatory period in the adult ovary (Molyneaux *et al.* 2003, Kryczek *et al.* 2005). Segers *et al.* analyzed the effects of gonadotropins on *Cxcr4* in theca cells during antral follicle development and suggested a synergy of *Lhcgr* and the FSH receptor (*Fshr)* in inducing *Cxcr4* expression (Segers *et al.* 2012). Interestingly, *Lhcgr* is upregulated in FL1 in diestrus (Table 3), which supports the hypothesis that an interaction of *Cxcr4* and the gonadotropin receptors, especially *Lhcgr*, plays a role in the increased ovulation rate of FL1 by promoting follicular survival. Besides the potential synergy with *Cxcr4*, the role of *Lhcgr* in folliculogenesis is well established, and a great number of activating or inactivation mutations and also knockout models have been described in literature (Hunzicker-Dunn & Mayo 2006). At this point, however, the question arises how an upregulation of *Lhcgr* in the ovary of FL1 can occur, since it is known to be a target gene of FSH, which is significantly decreased in FL1 throughout the whole estrous cycle. Interestingly, several studies suggest that signaling via cAMP (cyclic AMP) and AKT pathways is able to activate target genes of FSH (Zeleznik *et al.* 2003, Hunzicker-Dunn & Mayo 2006, Escamilla-Hernandez *et al.* 2008) and occupy a significant position in the function of FSH (Chu *et al.* 2018). This promotes the hypothesis that the decreased FSH levels in FL1 might be compensated by other factors able to activate intracellular pathways, which are actually induced by FSH. Due to the fact that LH is known to regulate follicular maturation via cAMP signaling (Hunzicker-Dunn & Mayo 2006), increased LH levels and *Lhcgr* expression rates in the ovaries of FL1 mice might be involved in this process. However, the regulation of intracellular signaling pathways is a complex cell-specific interplay of different endocrine and molecular factors and is dependent of the stage of follicular development of the follicles. We also should consider that non-genomic actions via membrane bound steroid hormone receptors might play a role in the increased reproductive performance of FL1 females. But since the ovaries used in this study consist of a variety of different developmental stages of follicles, which in turn contain various different cell types, an informative analysis of signaling pathways is not achievable and has to be analyzed in further studies.

**Figure 9:**
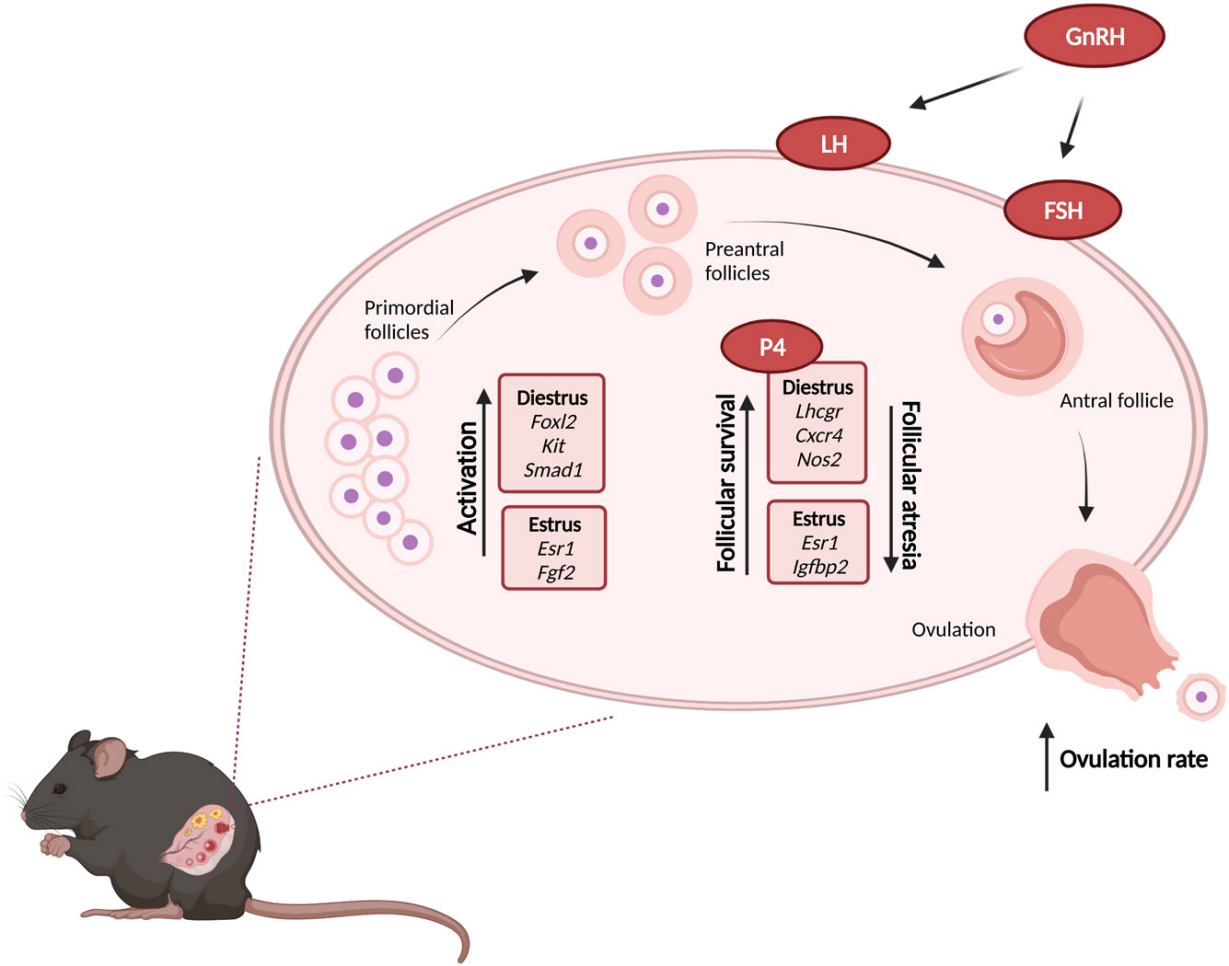
Follicular maturation in FL1 mice. Gene expression data and the measurement of parameters of the HPG axis on endocrine levels indicate that different traits of follicular development are improved in FL1 mice. Our results demonstrate that the increased ovulation rate of FL1 mice is due to a complex interplay of different cycle-related alterations, leading to increased activation of primordial follicles, decreased apoptosis, and increased follicular survival (figure created with BioRender.com). A full color version of this figure is available at https://doi.org/10.1530/JME-22-0012.

Taken together, our data indicate that different traits of follicular development are modified in FL1 mice. A graphical summary of the alterations in the different stages of follicular development of monovular follicles is given in Fig. 9. However, like discussed above, one should take in consideration the occurrence of POFs is characteristic for FL1 mice and they have the potential to ovulate more than one oocyte per follicle.

Another aspect of the increased reproductive fitness of FL1 is the reproductive lifespan. In both lines, ctrl and FL1, the reproductive success decreases with increasing age of the mice; however, this effect appears earlier in FL1 than in ctrl (Langhammer *et al.* 2021). *Per2*, which revealed lower levels in the estrus of FL1 vs ctrl mice, is a gene that is suggested to cause advanced ageing and therefore reproductive deficits. While the reproductive success of young *Per2^−/−^* mice does not differ from the wild-type, middle-aged *Per2^−/−^* mice are characterized by reproductive failures, that are comparable with those, which are normally observed in old WT mice (Pilorz & Steinlechner 2008). Nevertheless, FL1 is the mouse line with the highest fecundity index that is described in literature (Langhammer *et al.* 2021). *Tex14*, which is on a higher transcript level in the estrus of FL1 vs ctrl mice, is another gene that is involved in reproductive lifespan. Although adult *Tex14* knockout mice had normal-appearing viable oocytes and follicles at every stage of folliculogenesis and they were fertile, the *Tex14^−/−^* females delivered a significant lower number of litters over a 6 months period than the *Tex14^+/−^* females (Greenbaum *et al.* 2009). Although not experimentally tested, overexpression of *Tex14* might increase ovulation rates and play a role in the reproductive lifespan of FL1.

## Conclusions

Recapitulating the gene expression approach and hormonal measurements it is likely that the increased reproductive performance is due to a complex interplay of different cycle-related alterations. Our data indicate that several processes associated with folliculogenesis are modified in FL1 on molecular and endocrine levels to ensure high ovulation rates. Moreover, our results demonstrate that a low concentration/expression rate of FSH cannot be attributed to a low ovulation rate but has to be considered in connection with local mechanisms in the ovary and other endocrine parameters. However, to specify the results of this study, cell-specific signaling pathways have to be analyzed in future experiments. The Dummerstorf high fertility mouse line FL1 has developed a way not only to increase the ovulation rate but also to deliver high litter sizes over a long time period. Thus, this unique high-fertility model can provide new insights into many different aspects of female reproductive fitness and has the potential to improve our understanding of the achievement of high fertility and longevity.

## Supplementary materials

Supplementary file 2 Weight of reproductive organs of ctrl and FL1 mice in estrus and diestrus

Supplementary file 1 Primers for quantitative real-time PCR

Supplementary file 3 List of genes associated with reproductive traits in estrus and diestrus

## Declaration of interest

The authors declare that there is no conflict of interest that could be perceived as prejudicing the impartiality of the research reported.

## Funding

This work was supported by the German Research Foundation (DFG, MI 2098/3-1).

## Ethics approval and consent to participate

All procedures were performed following national and international guidelines and approved by the institutional board (Animal Protection Board from the Research Institute for Farm Animal Biology). The study is in accordance with ARRIVE guidelines.

## Availability of data and materials

The datasets used and/or analyzed during the current study are available from the corresponding author on reasonable request.

## Author contribution statement

C L M L, M M and J M W conceived the experimental design. M L was responsible for animal breeding. E K W organized the gonadotropin measurement. S B was responsible for the mRNA-Seq and conducted the statistical evaluation of the transcriptome data. C L performed the statistical evaluation of hormonal measurement and qPCR, interpreted the results, and drafted the manuscript. M T V created the bio RENDER figure. A R, U S, J M W and M M provided substantial suggestions on interpretation presentation of the data sets and critically read the manuscript. All authors read the manuscript and approved the final manuscript. The authors declare that there is no conflict of interest that could be perceived as prejudicing the impartiality of the research reported.
