# Supplementary material for: Endocrine and molecular factors of increased female reproductive performance in the Dummerstorf high-fertility mouse line FL1
Source: J Mol Endocrinol. 2022 Apr 6;69(1):285–98. doi: 10.1530/JME-22-0012 (PMC9175557; doi:10.1530/JME-22-0012)
Supplement: Supplementary file 2 Weight of reproductive organs of ctrl and FL1 mice in estrus and diestrus [file supplementary_table_1.pdf]

Supplementary file 1 Primers for quantitative real-time PCR

Gene	Primer sequence (5´-3´)	
	Forward	Reverse
GnRH	ATGATCCTCAAACTGATGGCCG	TTCTGCCATTTGATCCACCTCC
FSH	CAGTAGAGAAGGAAGAGTGCCG	TGTGTAGAGGGAGTCTGAGTGG
LH	AATGAGTTCTGCCCAGTCTGC	CACAGGCCATTGGTTGAGTCC
Igfbp2	AACATCTCTACTCCCTGCACATC	CTCGTTGTAGAAGAGATGGCACT
Per2	ACCTCCGAGTATATCGTGAAGAAC	TGCACTCCTGAGTGAAAGAATCTA
Esr1	CGCCTTCTACAGGTCTAATTCTGA	CACATGTAGTCATTGTGTCCTTGA
Agt	GACCTCCTGACTTGGATAGAGAAC	GTGTCACCAATGTTGTTCAGATTT
Kl	CTTTCAGCTATTGGACCCTAACAT	CCAGTCTGATTGCTTTTAAGGTTT
Fetub	CTCTGTTCTACCTCACATTGGATG	ATGAGTCTTTCTTTTGGAAACTGG
Kit	AAAAGATGTGTCTACATCCGTGAA	TGACATTTGCTGATCCAAAAGTAT
F2r	GATTGTGCACTACCTTTTCCTCTC	GGTGACTAGAGCAGGTATCCATTT
Foxl2	ACCAGTACATCATAGCCAAGTTCC	GGTAGTTGCCCTTCTCGAACAT
Klf2	ACACATACTTGCAGCTACACCAAC	ACTGAAAGGGTCTGTGACCTGT
Rora	TCAAAATGATCAAATTGTGCTTCT	AGAACACAAACTCTTCCCAAATTC
Smad1	GTACTATGAGCTCAACAACCGTGT	ACACCTCTCCTCCAACGTAATAAA
Lhcgr	CTCGGTTAAAATACCTGAGCATCT	GCTTTGTACTTCTTCAAACCCATT
Cxcr4	AGACTATGACTCCAACAAGGAACC	ATGCTCCTTAGCTTCTTCTGGTAA
RPS18	ACCATCATGCAGAACCCACGACAGT	CAGGTCCTCACGCAGCTTGTTGTCT
36B4	AAGCGCGTCCTGGCATTGTCT	CCGCAGGGGCAGCAGTGGT
B2m	TTCTGGTGCTTGTCTCACTGAC	GCAGTTCAGTATGTTCGGCTTC
GADPH	TCACCATCTTCCAGGAGCGAGAC	TTTCTCGTGGTTCACACCCATCA
