# Supplementary material for: Endocrine and molecular factors of increased female reproductive performance in the Dummerstorf high-fertility mouse line FL1
Source: J Mol Endocrinol. 2022 Apr 6;69(1):285–98. doi: 10.1530/JME-22-0012 (PMC9175557; doi:10.1530/JME-22-0012)
Supplement: Supplementary file 1 Primers for quantitative real-time PCR [file supplementary_table_2.pdf]

Supplementary file 2 Weight of reproductive organs of ctrl and FL1 mice in estrus and diestrus

	**Right ovary (g)**		**Left ovary (g)**		**Uterus (g)**	
	Estrus	Diestrus	Estrus	Diestrus	Estrus	Diestrus
ctrl	0.018 ± 0.0031	0.018 ± 0.0008	0.019 ± 0.0027	0.017 ± 0.0013	0.18 ± 0.013	0.11 ± 0.005
FL1	0.017 ± 0.0007	0.016 ± 0.0006	0.018 ± 0.0008	0.016 ± 0.0008	0.18 ± 0.008	0.13 ± 0.008
