# Supplementary material for: Endocrine and molecular factors of increased female reproductive performance in the Dummerstorf high-fertility mouse line FL1
Source: J Mol Endocrinol. 2022 Apr 6;69(1):285–98. doi: 10.1530/JME-22-0012 (PMC9175557; doi:10.1530/JME-22-0012)
Supplement: Supplementary file 3 List of genes associated with reproductive traits in estrus and diestrus [file supplementary_table_3.pdf]

Supplementary file 3 List of genes associated with reproductive traits in estrus and diestrus

Estrus

Gene	*ΔLog2FC*	*ΔLog2FC qPCR*	*keyword*
	Genes associated with fetal or embryonic growth and development		
Rpl29	*-5.3****		*Embryonic growth (1)*
Procr	*-1.1***		*Embryonic growth retardation, embryo size (2)*
Morf4l1	*1.3***		*Embryonic development (3)*
Dusp23	*1.5****		*Embryonic growth retardation (4)*
Hsd17b2	*1.5***		*Placental morphology and size, embryonic and fetal growth, birth weight (5)*
Zfp949	*1.6***		*Embryonic development (6)*
Spint1	*1.7**		*Abnormal placenta morphology and development (7); embryonic growth retardation (8)*
	Genes associated with the reproductive lifespan		
Per2	*-1.5**	*-1.4**	*Absent estrous cycle, decreased litter size, Reproductive lifespan (9)*
Kl	*1.6**	*1.6***	*Reproductive lifespan (10)*
Tex14	*2.1****		*Reproductive lifespan (11)*
	Genes associated with ovulation rate or folliculogenesis		
Hpgd	*-1.4****		*P_4 level, morphology of corpus luteum (12)*
Agt	*-1.2**	*-1.1***	*Ovulation rate (13)*
Peg3	*-1.1****		*Number of mature oocytes, litter size (14)*
Igfbp2	*-0.9***	*-0.8***	*Follicular development (15)*
Esr1	*-0.7**	*-0.7***	*Granulosa cell proliferation (16); folliculogenesis (17); alteration of LH-and FSH levels (18-20); estrous cycle (21); number of corpora lutea (19, 20); ovulation rate (22)*
	Genes associated with other traits of fertility		
Lepr	*-1.1**		*Fertility, estrous cycle (23); number of ovarian follicles (24)*
Efemp1	*-1.0***		*Litter size (25)*
Pcna	*-0.7***		*Absent germ cells (26)*
Asb4	*1.1**		*Vascularisation of placenta (27)*
Setd3	*0.8**		*Litter size (28)*

Trf	*0.8***		*Estrous cycle (29)*
Fetub	*1.7**	*1.6***	*Fertilization (30)*

The ΔLog2FC is calculated based on the result of the transcriptome analysis (* q < 0.05, ** q < 0.01, *** q < 0.001, n =5). Additionally the ΔLog2FC based on the results of the qPCR validation experiment is shown (n = 10)

Diestrus

Gene	*ΔLog2FC*	*ΔLog2FC qPCR*	*keyword*
	Genes associated with fetal or embryonic growth and development		
F2r	*-1.1***	*-0.9***	*Placental and embryonic development (31, 32); fetal development (33)*
Hspg2	*-0.6**		*Embryo size (34)*
Nfe2l1	*-0.5****		*Embryonic development (35, 36)*
Klf2	*0.6**	*-0.2*	*Embryonic growth (37)*
Klf3	*0.6****		*Embryonic growth and survival (38)*
C2	*0.7**		*Embryonic development (39)*
Ptges	*0.7**		*Embryonic growth (40)*
Tgfbr1	*0.7***		*Vascularisation of the placenta, embryo size (41-43)*
Slc30a1	*0.9****		*Embryonic development and growth (44)*
Slc20a1	*1.0***		*Embryonic growth (45)*
Folr1	*1.1****		*Embryonic development (46)*
Hsd17b7	*2.0**		*Embryo size (47)*
	Genes associate with reproductive lifespan		
Rora	*0.7**	*0.4**	*Estrous cycle, reproductive life span (48, 49); number of mature oocytes (50)*
	Genes associated with ovulation rate or folliculogenesis		
Gabrb1	*-1.7**		*Morphology of corpus luteum, levels of FSH, LH and PRL (51)*
Psmc3ip	*-1.4***		*Ovarian development, ovarian follicles (52)*
Kit	*-1.4**	*-1.5***	*Folliculogenesis (53); oocyte morphology (54); LH level (53); litter size (55); primordial cell migration (56-58); ovarian follicle number (55)*
Nos2	*-0.9***		*Germ cell morphology (59); litter size (60); morphology of the placenta (60); PRL level (61); estrous cycle, ovulation rate,*

			ovarian morphology, steroidogenesis (62), marker of follicular atresia (63)
Npc1	*-0.6****		*Morphology of granulosa cells, ovarian follicles and corpus luteum (64)*
Foxl2	*0.5***	*-0.2*	*Folliculogenesis (65); granulosa cell differentiation (66);*
Smad1	*0.8***	*1.4**	*Embryonic development (67); primordial germ cells (68-70); folliculogenesis (71)*
Fzd4	*0.9****		*Formation of corpus luteum (72)*
Lhcgr	*1.4**	*1.2***	*Folliculogenesis, formation of corpus luteum (73, 74); levels of FSH, LH, P_4 and E_2 (74);*
Cxcr4	*1.5****	*1.5**	*Primordial germ cell migration (75, 76)*
Genes associated with other traits of fertility			
Cxcl14	*-1.0****		*Litter size (77)*
Gsta4	*-0.8****		*Litter size (78)*
Net1	*-0.7**		*Mammary gland development (79)*
Ptprs	*0.5**		*Estrous cycle, alteration of LHRH expression (80)*
Kalrn	*1.1***		*PRL and GH secretion (81)*
Fetub	*1.3***	*1.6***	*Fertilization (30)*
Mmp12	*2.2****		*Litter size (82)*

The ΔLog2FC is calculated based on the result of the transcriptome analysis (* q < 0.05, ** q < 0.01, *** q < 0.001, n = 5). Additionally the ΔLog2FC based on the results of the qPCR validation experiment is shown (n = 10)

1. Kirn-Safran CB, Oristian DS, Focht RJ, Parker SG, Vivian JL, Carson DD. Global growth deficiencies in mice lacking the ribosomal protein HIP/RPL29. Developmental Dynamics. 2007;236(2):447-60.
2. Gu JM, Crawley JT, Ferrell G, Zhang F, Li W, Esmon NL, et al. Disruption of the endothelial cell protein C receptor gene in mice causes placental thrombosis and early embryonic lethality. The Journal of biological chemistry. 2002;277(45):43335-43.
3. Tominaga K, Kirtane B, Jackson JG, Ikeno Y, Ikeda T, Hawks C, et al. MRG15 regulates embryonic development and cell proliferation. Mol Cell Biol. 2005;25(8):2924-37.
4. Shen J, Liu X, Yu W-M, Liu J, Groot Nibbelink M, Guo C, et al. A Critical Role of Mitochondrial Phosphatase Ptpmt1 in Embryogenesis Reveals a Mitochondrial Metabolic Stress-Induced Differentiation Checkpoint in Embryonic Stem Cells. Molecular and Cellular Biology. 2011;31(24):4902-16.
5. Rantakari P, Strauss L, Kiviranta R, Lagerbohm H, Paviala J, Holopainen I, et al. Placenta Defects and Embryonic Lethality Resulting from Disruption of Mouse Hydroxysteroid (17-β) Dehydrogenase 2 Gene. Molecular Endocrinology. 2008;22(3):665-75.
6. Teratake Y, Kuga C, Hasegawa Y, Sato Y, Kitahashi M, Fujimura L, et al. Transcriptional repression of p27 is essential for murine embryonic development. Scientific Reports. 2016;6(1):26244.

7. Szabo R, Uzzun Sales K, Kosa P, Shylo NA, Godiksen S, Hansen KK, et al. Reduced Prostasin (CAP1/PRSS8) Activity Eliminates HAI-1 and HAI-2 Deficiency–Associated Developmental Defects by Preventing Matriptase Activation. PLOS Genetics. 2012;8(8):e1002937.
8. Szabo R, Molinolo A, List K, Bugge TH. Matriptase inhibition by hepatocyte growth factor activator inhibitor-1 is essential for placental development. Oncogene. 2007;26(11):1546-56.
9. Pilorz V, Steinlechner S. Low reproductive success in Per1 and Per2 mutant mouse females due to accelerated ageing? 2008;135(4):559.
10. Kuro-o M, Matsumura Y, Aizawa H, Kawaguchi H, Suga T, Utsugi T, et al. Mutation of the mouse klotho gene leads to a syndrome resembling ageing. Nature. 1997;390(6655):45-51.
11. Greenbaum MP, Iwamori N, Agno JE, Matzuk MM. Mouse TEX14 is required for embryonic germ cell intercellular bridges but not female fertility. Biol Reprod. 2009;80(3):449-57.
12. Roizen JD, Asada M, Tong M, Tai H-H, Muglia LJ. Preterm Birth without Progesterone Withdrawal in 15-Hydroxyprostaglandin Dehydrogenase Hypomorphic Mice. Molecular Endocrinology. 2008;22(1):105-12.
13. Hefler LA, Gregg AR. Influence of the angiotensinogen gene on the ovulatory capacity of mice. Fertility and sterility. 2001;75(6):1206-11.
14. Kim J, Frey WD, He H, Kim H, Ekram MB, Bakshi A, et al. Peg3 Mutational Effects on Reproduction and Placenta-Specific Gene Families. PLOS ONE. 2014;8(12):e83359.
15. Spitschak M, Hoeflich A. Potential Functions of IGFBP-2 for Ovarian Folliculogenesis and Steroidogenesis. Front Endocrinol (Lausanne). 2018;9:119-.
16. Dupont S, Krust A, Gansmuller A, Dierich A, Chambon P, Mark M. Effect of single and compound knockouts of estrogen receptors alpha (ERalpha) and beta (ERbeta) on mouse reproductive phenotypes. Development (Cambridge, England). 2000;127(19):4277-91.
17. Schomberg DW, Couse JF, Mukherjee A, Lubahn DB, Sar M, Mayo KE, et al. Targeted disruption of the estrogen receptor-alpha gene in female mice: characterization of ovarian responses and phenotype in the adult. Endocrinology. 1999;140(6):2733-44.
18. Adlanmerini M, Solinhac R, Abot A, Fabre A, Raymond-Letron I, Guihot A-L, et al. Mutation of the palmitoylation site of estrogen receptor α in vivo reveals tissue-specific roles for membrane versus nuclear actions. Proceedings of the National Academy of Sciences. 2014;111(2):E283-E90.
19. Lee S, Kang D-W, Hudgins-Spivey S, Krust A, Lee E-Y, Koo Y, et al. Theca-Specific Estrogen Receptor-α Knockout Mice Lose Fertility Prematurely. Endocrinology. 2009;150(8):3855-62.
20. Singh SP, Wolfe A, Ng Y, DiVall SA, Buggs C, Levine JE, et al. Impaired Estrogen Feedback and Infertility in Female Mice with Pituitary-Specific Deletion of Estrogen Receptor Alpha (ESR1)1. Biology of Reproduction. 2009;81(3):488-96.
21. Gieske MC, Kim HJ, Legan SJ, Koo Y, Krust A, Chambon P, et al. Pituitary Gonadotroph Estrogen Receptor-α Is Necessary for Fertility in Females. Endocrinology. 2008;149(1):20-7.
22. Jakacka M, Ito M, Martinson F, Ishikawa T, Lee EJ, Jameson JL. An estrogen receptor (ER)alpha deoxyribonucleic acid-binding domain knock-in mutation provides evidence for nonclassical ER pathway signaling in vivo. Mol Endocrinol. 2002;16(10):2188-201.
23. Johnson LM, Sidman RL. A Reproductive Endocrine Profile in the Diabetes (db) Mutant Mouse1. Biology of Reproduction. 1979;20(3):552-9.
24. Xia H, Zhang R, Guan H, Zhang W. Follicle loss and PTEN/PI3K/mTOR signaling pathway activated in LepR-mutated mice. Gynecol Endocrinol. 2019;35(1):44-8.
25. McLaughlin PJ, Bakall B, Choi J, Liu Z, Sasaki T, Davis EC, et al. Lack of fibulin-3 causes early aging and herniation, but not macular degeneration in mice. Human Molecular Genetics. 2007;16(24):3059-70.
26. Langerak P, Nygren AOH, Krijger PHL, van den Berk PCM, Jacobs H. A/T mutagenesis in hypermutated immunoglobulin genes strongly depends on PCNAK164 modification. Journal of Experimental Medicine. 2007;204(8):1989-98.
27. Townley-Tilson WHD, Wu Y, Ferguson JE, III, Patterson C. The Ubiquitin Ligase ASB4 Promotes Trophoblast Differentiation through the Degradation of ID2. PLOS ONE. 2014;9(2):e89451.
28. Wilkinson AW, Diep J, Dai S, Liu S, Ooi YS, Song D, et al. SETD3 is an actin histidine methyltransferase that prevents primary dystocia. Nature. 2019;565(7739):372-6.

29. Robertson SA, Mau VJ, Young IG, Matthaei KI. Uterine eosinophils and reproductive performance in interleukin 5-deficient mice. Journal of reproduction and fertility. 2000;120(2):423-32.
30. Dietzel E, Wessling J, Floehr J, Schäfer C, Ensslen S, Denecke B, et al. Fetuin-B, a Liver-Derived Plasma Protein Is Essential for Fertilization. Developmental Cell. 2013;25(1):106-12.
31. Connolly AJ, Ishihara H, Kahn ML, Farese RV, Coughlin SR. Role of the thrombin receptor in development and evidence for a second receptor. Nature. 1996;381(6582):516-9.
32. Griffin CT, Srinivasan Y, Zheng Y-W, Huang W, Coughlin SR. A Role for Thrombin Receptor Signaling in Endothelial Cells During Embryonic Development. Science (New York, NY). 2001;293(5535):1666-70.
33. Darrow AL, Fung-Leung WP, Ye RD, Santulli RJ, Cheung WM, Derian CK, et al. Biological consequences of thrombin receptor deficiency in mice. Thrombosis and haemostasis. 1996;76(6):860-6.
34. Gustafsson E, Aszodi A, Ortega N, Hunziker EB, Denker HW, Werb Z, et al. Role of collagen type II and perlecan in skeletal development. Annals of the New York Academy of Sciences. 2003;995:140-50.
35. Chan JY, Kwong M, Lu R, Chang J, Wang B, Yen TSB, et al. Targeted disruption of the ubiquitous CNC-bZIP transcription factor, Nrf-1, results in anemia and embryonic lethality in mice. The EMBO Journal. 1998;17(6):1779-87.
36. Leung L, Kwong M, Hou S, Lee C, Chan JY. Deficiency of the Nrf1 and Nrf2 transcription factors results in early embryonic lethality and severe oxidative stress. The Journal of biological chemistry. 2003;278(48):48021-9.
37. Wani MA, Means RT, Jr., Lingrel JB. Loss of LKLF function results in embryonic lethality in mice. Transgenic research. 1998;7(4):229-38.
38. Funnell APW, Mak KS, Twine NA, Pelka GJ, Norton LJ, Radziewic T, et al. Generation of Mice Deficient in both KLF3/BKLF and KLF8 Reveals a Genetic Interaction and a Role for These Factors in Embryonic Globin Gene Silencing. Molecular and Cellular Biology. 2013;33(15):2976-87.
39. Williamson DJ, Banik-Maiti S, DeGregori J, Ruley HE. hnRNP C is required for postimplantation mouse development but Is dispensable for cell viability. Mol Cell Biol. 2000;20(11):4094-105.
40. Nakatani Y, Hokonohara Y, Kakuta S, Sudo K, Iwakura Y, Kudo I. Knockout mice lacking cPGES/p23, a constitutively expressed PGE2 synthetic enzyme, are peri-natally lethal. Biochemical and Biophysical Research Communications. 2007;362(2):387-92.
41. Seki T, Hong K-H, Oh SP. Nonoverlapping expression patterns of ALK1 and ALK5 reveal distinct roles of each receptor in vascular development. Laboratory Investigation. 2006;86(2):116-29.
42. Carvalho RLC, Itoh F, Goumans M-J, Lebrin F, Kato M, Takahashi S, et al. Compensatory signalling induced in the yolk sac vasculature by deletion of TGFβ receptors in mice. Journal of Cell Science. 2007;120(24):4269-77.
43. Larsson J, Goumans M-J, Sjöstrand LJ, van Rooijen MA, Ward D, Levéen P, et al. Abnormal angiogenesis but intact hematopoietic potential in TGF-β type I receptor-deficient mice. The EMBO Journal. 2001;20(7):1663-73.
44. Andrews GK, Wang H, Dey SK, Palmiter RD. Mouse zinc transporter 1 gene provides an essential function during early embryonic development. genesis. 2004;40(2):74-81.
45. Festing MH, Speer MY, Yang H-Y, Giachelli CM. Generation of mouse conditional and null alleles of the type III sodium-dependent phosphate cotransporter PiT-1. genesis. 2009;47(12):858-63.
46. Piedrahita JA, Oetama B, Bennett GD, van Waes J, Kamen BA, Richardson J, et al. Mice lacking the folic acid-binding protein Folbp1 are defective in early embryonic development. Nature genetics. 1999;23(2):228-32.
47. Shehu A, Mao J, Gibori GB, Halperin J, Le J, Sangeeta Devi Y, et al. Prolactin Receptor-Associated Protein/17β-Hydroxysteroid Dehydrogenase Type 7 Gene (Hsd17b7) Plays a Crucial Role in Embryonic Development and Fetal Survival. Molecular Endocrinology. 2008;22(10):2268-77.
48. Guastavino JM, Larsson K. The staggerer gene curtails the reproductive life span of females. Behavior genetics. 1992;22(1):101-12.

49. Guastavino JM, Larsson K, Allain C, Jaisson P. Neonatal vestibular stimulation and mating in cerebellar mutants. Behavior genetics. 1993;23(3):265-9.
50. Guastavino JM, Boufares S, Crusio WE. Ovarian abnormalities in the staggerer mutant mouse. ScientificWorldJournal. 2005;5:661-4.
51. Anstee QM, Knapp S, Maguire EP, Hosie AM, Thomas P, Mortensen M, et al. Mutations in the Gabrb1 gene promote alcohol consumption through increased tonic inhibition. Nature Communications. 2013;4(1):2816.
52. Petukhova GV, Romanienko PJ, Camerini-Otero RD. The Hop2 protein has a direct role in promoting interhomolog interactions during mouse meiosis. Dev Cell. 2003;5(6):927-36.
53. Kissel H, Timokhina I, Hardy MP, Rothschild G, Tajima Y, Soares V, et al. Point mutation in Kit receptor tyrosine kinase reveals essential roles for Kit signaling in spermatogenesis and oogenesis without affecting other Kit responses. The EMBO Journal. 2000;19(6):1312-26.
54. Reynaud K, Cortvrindt R, Smitz J, Bernex F, Panthier JJ, Driancourt MA. Alterations in ovarian function of mice with reduced amounts of KIT receptor. Reproduction (Cambridge, England). 2001;121(2):229-37.
55. Geissler EN, McFarland EC, Russell ES. Analysis of pleiotropism at the dominant white-spotting (W) locus of the house mouse: a description of ten new W alleles. Genetics. 1981;97(2):337-61.
56. Bernex F, De Sepulveda P, Kress C, Elbaz C, Delouis C, Panthier JJ. Spatial and temporal patterns of c-kit-expressing cells in WlacZ/+ and WlacZ/WlacZ mouse embryos. Development (Cambridge, England). 1996;122(10):3023-33.
57. Buehr M, McLaren A, Bartley A, Darling S. Proliferation and migration of primordial germ cells in We/We mouse embryos. Developmental Dynamics. 1993;198(3):182-9.
58. Wu BJ, Yin LJ, Yin HP, Ying XS, Yang WW, Zeng YM, et al. A mutation in the Kit gene leads to novel gonadal phenotypes in both heterozygous and homozygous mice. Hereditas. 2010;147(2):62-9.
59. Suzuki A, Saga Y. Nanos2 suppresses meiosis and promotes male germ cell differentiation. Genes & development. 2008;22(4):430-5.
60. Burnett TG, Tash JS, Hunt JS. Investigation of the role of nitric oxide synthase 2 in pregnancy using mutant mice. Reproduction (Cambridge, England). 2002;124(1):49-57.
61. Zaragozá R, Bosch A, García C, Sandoval J, Serna E, Torres L, et al. Nitric oxide triggers mammary gland involution after weaning: remodelling is delayed but not impaired in mice lacking inducible nitric oxide synthase. Biochemical Journal. 2010;428(3):451-62.
62. Jablonka-Shariff A, Ravi S, Beltsos AN, Murphy LL, Olson LM. Abnormal estrous cyclicity after disruption of endothelial and inducible nitric oxide synthase in mice. Biology of reproduction. 1999;61(1):171-7.
63. Nath P, Maitra S. Physiological relevance of nitric oxide in ovarian functions: An overview. Gen Comp Endocrinol. 2019;279:35-44.
64. Gévry NY, Lopes FL, Ledoux S, Murphy BD. Aberrant Intracellular Cholesterol Transport Disrupts Pituitary and Ovarian Function. Molecular Endocrinology. 2004;18(7):1778-86.
65. Uda M, Ottolenghi C, Crisponi L, Garcia JE, Deiana M, Kimber W, et al. Foxl2 disruption causes mouse ovarian failure by pervasive blockage of follicle development. Human Molecular Genetics. 2004;13(11):1171-81.
66. Schmidt D, Ovitt CE, Anlag K, Fehsenfeld S, Gredsted L, Treier A-C, et al. The murine winged-helix transcription factor Foxl2 is required for granulosa cell differentiation and ovary maintenance. Development (Cambridge, England). 2004;131(4):933-42.
67. Arnold SJ, Maretto S, Islam A, Bikoff EK, Robertson EJ. Dose-dependent Smad1, Smad5 and Smad8 signaling in the early mouse embryo. Developmental Biology. 2006;296(1):104-18.
68. Aubin J, Davy A, Soriano P. In vivo convergence of BMP and MAPK signaling pathways: impact of differential Smad1 phosphorylation on development and homeostasis. Genes & development. 2004;18(12):1482-94.
69. Tremblay KD, Dunn NR, Robertson EJ. Mouse embryos lacking Smad1 signals display defects in extra-embryonic tissues and germ cell formation. Development (Cambridge, England). 2001;128(18):3609-21.

70. Hayashi K, Kobayashi T, Umino T, Goitsuka R, Matsui Y, Kitamura D. SMAD1 signaling is critical for initial commitment of germ cell lineage from mouse epiblast. Mechanisms of development. 2002;118(1-2):99-109.
71. Pangas SA. Bone morphogenetic protein signaling transcription factor (SMAD) function in granulosa cells. Mol Cell Endocrinol. 2012;356(1-2):40-7.
72. Hsieh M, Boerboom D, Shimada M, Lo Y, Parlow AF, Luhmann UFO, et al. Mice Null for Frizzled4 (Fzd4−/−) Are Infertile and Exhibit Impaired Corpora Lutea Formation and Function1. Biology of Reproduction. 2005;73(6):1135-46.
73. Zhang FP, Poutanen M, Wilbertz J, Huhtaniemi I. Normal prenatal but arrested postnatal sexual development of luteinizing hormone receptor knockout (LuRKO) mice. Mol Endocrinol. 2001;15(1):172-83.
74. Lei ZM, Mishra S, Zou W, Xu B, Foltz M, Li X, et al. Targeted disruption of luteinizing hormone/human chorionic gonadotropin receptor gene. Mol Endocrinol. 2001;15(1):184-200.
75. Ara T, Nakamura Y, Egawa T, Sugiyama T, Abe K, Kishimoto T, et al. Impaired colonization of the gonads by primordial germ cells in mice lacking a chemokine, stromal cell-derived factor-1 (SDF-1). Proceedings of the National Academy of Sciences. 2003;100(9):5319-23.
76. Molyneaux KA, Zinszner H, Kunwar PS, Schaible K, Stebler J, Sunshine MJ, et al. The chemokine SDF1/CXCL12 and its receptor CXCR4 regulate mouse germ cell migration and survival. Development (Cambridge, England). 2003;130(18):4279-86.
77. Meuter S, Schaerli P, Roos RS, Brandau O, Bösl MR, von Andrian UH, et al. Murine CXCL14 Is Dispensable for Dendritic Cell Function and Localization within Peripheral Tissues. Molecular and Cellular Biology. 2007;27(3):983-92.
78. Engle MR, Singh SP, Czernik PJ, Gaddy D, Montague DC, Ceci JD, et al. Physiological role of mGSTA4-4, a glutathione S-transferase metabolizing 4-hydroxynonenal: generation and analysis of mGsta4 null mouse. Toxicology and Applied Pharmacology. 2004;194(3):296-308.
79. Zuo Y, Berdeaux R, Frost JA. The RhoGEF Net1 Is Required for Normal Mammary Gland Development. Molecular Endocrinology. 2014;28(12):1948-60.
80. Elchebly M, Wagner J, Kennedy TE, Lanctôt C, Michaliszyn E, Itié A, et al. Neuroendocrine dysplasia in mice lacking protein tyrosine phosphatase σ. Nature genetics. 1999;21(3):330-3.
81. Mandela P, Yankova M, Conti LH, Ma X-M, Grady J, Eipper BA, et al. Kalrnplays key roles within and outside of the nervous system. BMC Neuroscience. 2012;13(1):136.
82. Shipley JM, Wesselschmidt RL, Kobayashi DK, Ley TJ, Shapiro SD. Metalloelastase is required for macrophage-mediated proteolysis and matrix invasion in mice. Proc Natl Acad Sci U S A. 1996;93(9):3942-6.
